# Successful treatment of a kidney transplant patient with COVID-19 and late-onset *Pneumocystis jirovecii* pneumonia

**DOI:** 10.1186/s12941-021-00489-w

**Published:** 2021-12-15

**Authors:** Jing Peng, Ming Ni, Dunfeng Du, Yanjun Lu, Juan Song, Weiyong Liu, Na Shen, Xiong Wang, Yaowu Zhu, Bruce A. Vallance, Ziyong Sun, Hong Bing Yu

**Affiliations:** 1 Department of Laboratory Medicine, Tongji Hospital, Tongji Medical College, Huazhong University of Science and Technology, Wuhan, China; 2 Department of Infectious Diseases, Tongji Hospital, Tongji Medical College, Huazhong University of Science and Technology, Wuhan, China; 3 Institute of Organ Transplantation, Tongji Hospital, Tongji Medical College, Huazhong University of Science and Technology, Wuhan, China; 4 Department of Gastroenterology & Endocrinology, Wuhan No. 9 Hospital, Wuhan, China; 5 Department of Pediatrics, BC Children’s Hospital Research Institute, University of British Columbia, Vancouver, Canada

**Keywords:** COVID-19, PJP, *Pneumocystis jirovecii*, Kidney transplant patient

## Abstract

**Background:**

Solid transplant patients are susceptible to *Pneumocystis jirovecii* pneumonia (PJP). While the vast majority of PJP cases occur within the first 6 months after transplantation, very few PJP cases are seen beyond 1 year post-transplantation (late-onset PJP). PJP and coronavirus disease 2019 (COVID-19, caused by infection with SARS-CoV-2) share quite a few common clinical manifestations and imaging findings, making the diagnosis of PJP often underappreciated during the current COVID-19 pandemic. To date, only 1 case of kidney transplantation who developed COVID-19 and late-onset PJP has been reported, but this patient also suffered from many other infections and died from respiratory failure and multiple organ dysfunction syndrome. A successful treatment of kidney patients with COVID-19 and late-onset PJP has not been reported.

**Case presentation:**

We present a case of a 55-year-old male kidney transplant patient with COVID-19 who also developed late-onset PJP. He received a combined treatment strategy, including specific anti-pneumocystis therapy, symptomatic supportive therapy, adjusted immunosuppressive therapy, and use of antiviral drugs/antibiotics, ending with a favorable outcome.

**Conclusions:**

This case highlights the importance of prompt and differential diagnosis of PJP in kidney transplant patients with SARS-CoV-2 infection. Further studies are required to clarify if kidney transplant patients with COVID-19 could be prone to develop late-onset PJP and how these patients should be treated.

## Introduction

*Pneumocystis jirovecii* pneumonia (PJP), caused by the unicellular fungus *Pneumocystis jirovecii*, is increasingly seen in susceptible non-HIV-infected patients receiving solid organ transplants [1]. In solid organ transplant patients, the vast majority of PJP cases occur within the first 6 months after transplantation, whereas very few PJP cases are seen beyond 1 year post-transplantation [2]. Even so, Late-onset PJP remains as a major graft- and life-threatening complication in kidney transplant patients [3].

PJP shares common clinical presentations and imaging findings with coronavirus disease 2019 (COVID-19), a disease caused by severe acute respiratory syndrome coronavirus 2 (SARS-CoV-2). Both diseases can cause fever, nonproductive cough, shortness of breath, lymphocytopenia, and interstitial pneumonia [1, 4]. Since a growing body of evidence has indicated the high risk of COVID-19 patients for co-infections [5], it is important to identify if these patients suffer from additional infections, including *P. jirovecii* infection. We report a case of late-onset (6 years post transplantation) PJP in a kidney transplant patient with COVID-19. This patient was successfully treated with a combined strategy. We also review current English literatures following the case presentation.

## Case presentation

**Fig. 1:**
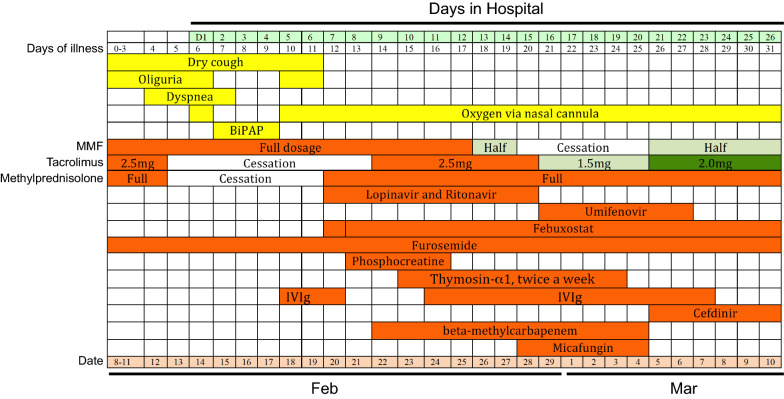
Diagram showing clinical symptoms and treatments before and after hospitalization

A 55-year-old male, with a history of chronic kidney disease for many years, received a living kidney transplant in 2013 and thereafter followed a regular immunosuppressive regimen, including mycophenolate mofetil (MMF) (0.5 g twice daily), tacrolimus (2 mg twice daily, the concentration was maintained at 4–5 ng/mL.), and methylprednisolone (5 mg once daily) (Fig. 1). His creatinine levels were maintained at around 100 μmol/L most of the time, but increased to 180 μmol/L on Jan 15, 2020. He used to experience shortness of breath on exertion. He also had a history of heart failure, regularly taking oral medications, including clopidogrel (75 mg once daily) and aspirin (100 mg once daily), and occasionally taking furosemide (60 mg once daily).

The patient presented to a local clinic in Wuhan on Jan 31, 2020, complaining of oliguria for two weeks. Chest computed tomography (CT) revealed bilateral lung lesions, with a lot of speckle consolidation shadows seen in both lungs. An oropharyngeal swab from this patient was positive for SARS-CoV-2 as shown by the reverse transcription-polymerase chain reaction (RT-PCR) assay, suggesting that he suffered from pneumonia caused by SARS-CoV-2. He displayed no fever, cough, dyspnea, diarrhea or vomiting at that time. He was then isolated in a makeshift hospital for further treatment.

**Fig. 2:**
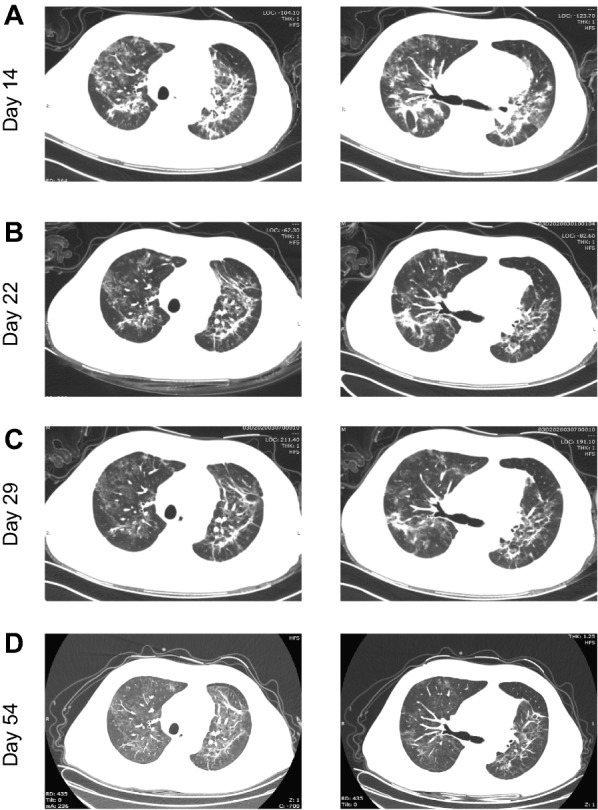
High-resolution computed tomography images at different days of illness. **A** Multiple patchy and stripe ground glass opacities near the hilum of bilateral lungs. **B** Absorption of ground-glass lesions compared to **A**. **C** Further absorption of ground-glass lesions compared to **B**. **D** Moderate absorption of ground-glass lesions compared to **C**

On Feb 8 (Day 0 of the illness, Day 0), the patient showed a mild cough without sputum, but developed dyspnea by Feb 12 (Day 4). He was then transferred to an intensive care unit in Tongji Hospital of Huazhong University of Science and Technology (Wuhan, China), a site designated for treating severe and critically ill COVID-19 patients on Feb 13 (Day 5). On admission, he had a blood pressure of 90/60 mm Hg, a pulse of 104 beats per minute, and an oxygen saturation of 94% while breathing ambient air. His laboratory testing showed lymphocytopenia, hypercreatinemia, and increased levels of D-dimers, C-reactive protein (CRP), and cardiac troponin I (Table 1). Moreover, his CT images showed bilateral lung interstitial disease, especially near the hilum, with ground-glass opacities (GGO) (Fig. 2a). A combined strategy was adopted to treat him, including cessation of tacrolimus and methylprednisolone, and initial supportive treatments (oxygen delivery via nasal cannula, furosemide therapy and injection of thymosin α1 and immunoglobulin) (Fig. 1). Thymosin α1 is a peptide originally isolated from thymic tissue, and shown to regulate immune function in many diseases, such as sepsis and chemotherapy-induced immunosuppression [6]. The patient responded well to the therapy.

**Table 1:** Clinical laboratory findings

Variable	Reference range	Hospital day 1 (day 6 of the illness)	Hospital day 8 (day 13 of the illness)	Hospital day 16 (day 21 of the illness)	Hospital day 23 (day 28 of the illness)	Day 55 of the illness
White blood cells (× 10^9^/L)	3.5–9.5	9.93	7.08	4.81	3.24	5.16
Total neutrophils (× 10^9^/L)	1.8–6.3	9.22	6.2	3.53	2.73	3.36
Total lymphocytes (× 10^9^/L)	1.1–3.2	0.39	0.31	0.63	0.31	1.24
CD4^+^ T (#/µL)	550–1440	–	258	292	72	-
Platelet count (× 10^9^/L)	125–350	172	115	174	112	99
Hemoglobin (g/L)	130–175	144	109	104	92	115
Alanine aminotransferase (U/L)	≤ 41	20	–	22	–	–
Aspartate aminotransferase (U/L)	≤ 40	33	–	25	–	–
Blood glucose	4.11–6.05	6.08	6.42	5.46	8.84	–
Lactate dehydrogenase (U/L)	135–225	–	443	465	245	264
Blood urea nitrogen (mmol/L)	3.6–9.5	38.7	–	17.8	–	15.1
Blood creatinine (µmol/L)	59–104	233	–	145	–	112
Blood uric acid (µmol/L)	202–416	644	–	262	–	422
eGFR (ml/min/liter)	> 90	26.1	–	46.4	–	63.4
High-sensitivity C-reactive protein (mg/L)	< 1.0	–	34.6	–	5.9	–
Procalcitonin (ng/ml)	0.02–0.05	–	0.14	0.09	0.16	0.07
Serum ferritin (µg/L)	30–400	–	1598.0	–	553.1	208.0
D-dimer (µg/ml)	< 0.5	–	2.27	–	0.49	–
Creatine kinase (U/L)	≤ 7.2	–	12.1	–	3.1	–
High-sensitivity cardiac troponin I (pg/ml)	≤ 34.2	296.2	388.2	–	79.0	–
IL-1β (pg/ml)	< 5.0	–	< 5.0	–	< 5.0	< 5.0
IL-2R (U/ml)	223–710	–	917	–	791	466
IL-6 (pg/ml)	< 7.0	–	31.83	–	< 1.50	2.46
IL-8 (pg/ml)	< 62	–	5.4	–	8.0	8.6
IL-10 (pg/ml)	< 9.1	–	< 5.0	–	< 5.0	< 5.0
TNF-α (pg/ml)	< 8.1	–	17.4	–	15.2	15.2

On Feb 20 (Day 12), the patient showed improved clinical symptoms (BP 118/81 mm Hg, HR 84 bpm, RR 16 bpm; the patient displayed good consciousness and spirit and showed no obvious cough, expectoration, fever, chill, abdominal pain, or diarrhea; the patient had a measured SpO2 of 100% when receiving 4 L/min oxygen through a nasal cannula) and was transferred to a general ward. Due to incomplete improvement in imaging results, high levels of CRP (34.6 mg/L) and procalcitonin (0.14 ng/mL) (Table 1), as well as a positive SARS-CoV-2 RNA test on an oropharyngeal swab, the patient received anti-bacterial and anti-viral treatment with beta-methylcarbapenem (0.3 g twice daily) and oral lopinavir/ritonavir (1000 mg/d), respectively. During this anti-bacterial and anti-viral therapy period, methylprednisolone (20 mg once daily), gradually reduced MMF dosage, and an adjusted dosage of tacrolimus, were also re-introduced (Fig. 1). While the patient subsequently showed improved clinical symptoms, his chest CT images on Feb 24 (Day 11) still showed extensive GGO (images not shown).

To identify potential respiratory pathogens associated with the abnormal image findings, several laboratory tests were ordered. On Feb 25 (Day 17), his oropharyngeal swab remained positive for SARS-CoV-2. Microscopic analysis of his expectorated sputum (also Feb 25) did not detect *P. jirovecii* or *Mycobacterium tuberculosis*. The result of his HIV test was negative. On Feb 28 (Day 20), his serum (1, 3)-beta-D glucan (BDG) level was 89.3 pg/mL, slightly above the normal range (< 70 pg/mL). He was suspected of carrying a fungal infection, especially PJP, based on the positive BDG test, his chest CT features and long immunosuppressive state. However, his impaired kidney function (with high creatinine level) at that time precluded empiric treatment with trimethoprim/sulfamethoxazole (TMP-SMX). Instead, he was treated with micafungin (50 mg IV gtt once daily), an echinocandin that inhibits the synthesis of BDG [7]. Additionally, the anti-bacterial regimen with beta-methylcarbapenem was maintained empirically. Considering the possibility of lopinavir/ritonavir in increasing the concentration of tacrolimus in his blood, anti-viral treatment was replaced with umifenovir (0.2 g three times daily). After one week of micafungin treatment, serum BDG levels fell within the normal range (61.61 pg/mL). His clinical symptoms and laboratory findings continued to improve (Table 1), with chest CT images on Mar 1 (Day 22) and Mar 8 (Day 29) showing gradual absorption of the ground-glass lesions (Fig. 2b and c). With his oropharyngeal swabs being SARS-CoV-2 negative on Mar 4 (Day 24) and 7 (Day 27), he was discharged on Mar 11 (Day 32). Since then, MMF was discontinued, but methylprednisolone (20 mg once daily) and tacrolimus (1 mg twice daily, the drug concentration was maintained at 2-3 ng/ml) were maintained. He remained well as of April 2 (Day 54), with lower serum creatinine level (112 μmol/L) (Table 1) and moderately improved chest CT images (Fig. 2d).

Given the incomplete absorption of the lesions in the lung but nearly normal serum creatinine level (112 μmol/L), we retrospectively analyzed the patient’s sputum collected on Feb 25 for the presence of *P. jirovecii* by a more sensitive method. Using a laboratory-developed quantitative real-time polymerase chain reaction (qPCR) assay that targets the mitochondrial large subunit ribosomal RNA gene of *P. jirovecii* [8], we found that the levels of *P. jirovecii* in his sputum were almost tenfold higher than those in a PJP positive sputum (collected from a non-COVID-19 patient). This finding suggested that the patient probably suffered from PJP. Accordingly, the patient received one month of anti-PJP treatment with TMP-SMX starting on April 4 (0.96 g twice daily), resulting in a favorable outcome, including significantly improved symptoms and imaging.

## Discussion

The first case of co-infection with SARS-CoV-2 and *P. jirovecii* was reported in a woman with hypoxemic respiratory failure [9]. She showed significantly increased serum BDG levels, highly positive PCR test, and was responsive to TMP-SMX treatment. Since then, more studies have revealed the increased incidence of co-infection of SARS-CoV-2 and *P. jirovecii* in immunocompromised patients (primarily in patients with HIV) [10–15]. Interestingly, the first case of PJP in an immunocompetent patient who recovered from COVID-19 pneumonia was also reported [16]. Despite these reports, there have been no studies estimating the incidence of concurrent COVID-19 and PJP in non-HIV patients who receive kidney transplantation and are often immunocompromised. To date, only 1 case of kidney transplantation who developed COVID-19 and late-onset PJP has been described. However, this patient also suffered from many other infections, such as CMV and *Aspergillosis*, and eventually died from respiratory failure and multiple organ dysfunction syndrome [17].

In the present case, the patient also suffered from both COVID-19 and late-onset PJP. He partially recovered from a combined treatment strategy as reported by others [18, 19], such as symptomatic supportive therapy, adjusted immunosuppressive therapy, and use of antiviral drugs/antibiotics. This combined treatment strategy may have partially rebuilt the host’s immune system, limiting the progression of PJP. However, his chest CT images on Feb 22 (Day 14) still showed extensive GGO, indicating an unresolved PJP. He was initially treated with micafungin. While micafungin is an echinocandin exhibiting potent activity against key pathogenic fungi, such as *Candida* and *Aspergillus*, it is not a therapeutic option against PJP [7, 20, 21]. There is very limited data on the use of caspofungin, another echinocandin, as a salvage therapy in PJP [22]. Indeed, no single echinocandin treatment should be used as a mono-therapy in PJP [21]. In line with this, this patient’s CT images had not improved until a period of treatment with micafungin and TMP-SMX was completed, indicating the effectiveness of these therapies specifically targeting *P. jirovecii* in this patient. It should be noted that the patient reported in our study showed much lower LDH, D-dimer, and serum creatinine levels as compared to the patient reported by De Francesco et al. [17] during his treatment, potentially contributing to his better recovery from the co-infection.

Due to the long-term use of immunosuppressive drugs, kidney transplant patients are susceptible to COVID-19 and PJP. This patient did not have a history of PJP. Although no obvious clinical symptoms were shown during his second visit, the slow resolution of his chest CT abnormalities suggests he may have had a late-onset of an atypical presentation of PJP as reported by others [23, 24]. While the optimal diagnosis of PJP relies on a combination of tests, including immunofluorescence microscopy, PCR and a BDG test [1], the sensitivities and susceptibilities of these tests vary depending on the types of specimens and the patient’s HIV status. Immunofluorescence microscopy remains the gold standard for PJP diagnosis, but lower pathogen burdens in HIV-negative patients could reduce its sensitivity [1]. This patient was HIV-negative. BDG concentrations are usually high in PJP patients. However, BDG is not specific for *P. jirovecii*, and some PJP patients exhibit low positive BDG concentrations [25]. Similar to this, the BDG levels in this patient were slightly higher than the normal range. The sensitivity of *P. jirovecii* PCR is very high [8], although this may raise concerns over the detection of *Pneumocystis* colonization. Based on the updated EORTC/MSGERC consensus definitions [26], the diagnosis of a proven PJP relies on both clinical and radiologic criteria plus demonstration of *P. jirovecii* by microscopy using conventional or immunofluorescence staining of tissue or respiratory tract specimens. The present case report showed a highly positive PCR test, low positive BDG concentrations, typical CT images, and the response to TMP-SMX therapy, but failed to demonstrate *P. jirovecii* by microscopy. We therefore concluded this case as being a probable PJP.

The patient’s PJP episode appeared to have a temporal relationship with COVID-19, but whether the co-infection of SARS-CoV-2 and *P. jirovecii* was more than a coincidence remains unclear. A recent study found an unexpectedly high proportion (9.3%) of critically ill COVID-19 patients were co-infected with *P. jirovecii* [10]. Interestingly, BDG concentrations in four out of five patients co-infected with *P. jirovecii* were low (< 120 pg/ml), similar to that seen in the patient reported here. The low concentration of BDG in patients from our study and that of Alanio et al. [10] suggest that these patients were probably colonized, but not infected per se, by *P. jirovecii*. Other studies also showed the co-infection of SARS-CoV-2 and *P. jirovecii* in patients with or without HIV infection [9, 11–14]. It is possible that SARS-CoV-2 infection further dampened the weakened immune system of this patient (who had already received immunosuppressive therapy), leading to heightened susceptibility to PJP. However, this hypothesis awaits further investigation. Intriguingly, CMV infection, together with intensive immunosuppression, are known to be significant risk factors for *Pneumocystis* infections [27, 28].

## Conclusions

We report a case of kidney transplant patient who developed both COVID-19 and late-onset PJP. He was successfully treated with a combined strategy, including specific anti-pneumocystis therapy, symptomatic supportive therapy, adjusted immunosuppressive therapy, and use of antiviral drugs/antibiotics, ending with a favorable outcome. More studies are required to clarify if kidney transplant patients with COVID-19 could be prone to develop late-onset PJP and how they should be treated. Another limitation of this study is that we cannot define this case as a proven PJP, as we were unable to detect *P. jirovecii* by microscopy in tissue or respiratory tract specimens. This is different from the first case of SARS-CoV-2 and *P. jirovecii* co-infection, which was diagnosed through histological identification of *Pneumocystis* cystic forms during the patient’s autopsy [29]. During the current COVID-19 pandemic, while it might be the priority to diagnose COVID-19 in transplant patients, special attention should also be paid to the prompt and differential diagnosis of PJP, particularly if they showed CT images typically seen in PJP. In cases where COVID-19 patients are strongly suspected of PJP and exhibit normal kidney function, a prophylactic treatment of PJP is also recommended.

## Data Availability

The datasets supporting the conclusions of this article are included within the article.

## Abbreviations

PJP: *Pneumocystis jirovecii* pneumonia
COVID-19: Coronavirus disease 2019
MMF: Mycophenolate mofetil
RT-PCR: Reverse transcription-polymerase chain reaction
CRP: C-reactive protein
GGO: Ground-glass opacities
BDG: (1, 3)-Beta-d glucan
